# The cephalic vein access for use in intensive care unit: an alternative approach

**DOI:** 10.1186/s13054-023-04498-3

**Published:** 2023-05-30

**Authors:** Shouyin Jiang, Yehua Shen, Xiaogang Zhao

**Affiliations:** 1 Department of Emergency Medicine, Second Affiliated Hospital, Zhejiang University School of Medicine, Key Laboratory of The Diagnosis and Treatment of Severe Trauma and Burn of Zhejiang Province, Zhejiang Province Clinical Research Center for Emergency and Critical Care Medicine, Research Institute of Emergency Medicine, Zhejiang University, Hangzhou, 310009 China; 2 Department of Radiology, The Children’s Hospital, Zhejiang University School of Medicine, National Clinical Research Center for Child Heath, Hangzhou, China

## Dear Editor,

We thank Gawda and Czarnik for their interest in our report introducing the cephalic vein access in intensive care unit (ICU) for assisting hemodynamic monitoring and vasoactive support in a special condition [1]. They have raised four questions with regard to the reason for central venous catheter (CVC) replacement, optional cannulation site, risk of thrombotic complications, and the definition of obesity.

As everyone knows, it is important for severe trauma patients to establish an ideal location for central venous cannulation. Patients who have prolonged ICU length of stay may require multiple cannulations. Factors influencing the choice of cannulation site can include coagulation disorders, thrombocytopenia, skin condition, subcutaneous tissue thickness, state of or expected tracheotomy, and so on. Although it is not recommended to remove a CVC regularly, suspicion of catheter associated infection, catheter occlusion, local infection or catheter displacement are factors to consider for catheter replacement for patients who still require CVC support. For our patient, decision for replacement of the central venous catheter was based on suspicion of bloodstream infection while she should require vasopressor support (norepinephrine 0.13 μg/kg/min) and invasive hemodynamic management. In our patient, the thick and edematous subcutaneous soft tissue (as showing in figure) made it difficult to choose an axillary vein route due to anticipated cannulation difficulty, although we are experienced in this procedure as we have practiced that for over 200 cases since 2021. Moreover, the state of tracheotomy and the short neck did not allow us to choose the internal jugular vein, this route may be associated with increased risk of catheter-related thrombosis (CRT) and line displacement, even with increased risk of injury to the common carotid artery.

We acknowledge the importance of the catheter to vessel ratio in real condition due to consideration of CRT [2]. A prospective multicenter study conducted in 28 intensive care units identified 16.9% incidence of CRT, with the internal jugular vein being the most common CRT found in the study [3]. However, the pathogenesis of CRT is multifactorial and complicated, with risk factors associated with the catheter per se, the vessel selected and the underlying co-morbidities and related treatments. Anatomically, the cephalic vein is approximately 6 mm in diameter [4]. Moreover, the measured diameter of the vein selected under ultrasound does not equal to the actual diameter, which can be influenced by volume state and vascular tension. According to the finding from our random ultrasound scanning, the diameter of cephalic vein in some patients would exceed 3 or even 4 mm, sometimes it could also be invisible. A rational catheter management and antithrombotic strategies (such as ultrasound screening and refined anticoagulant plan) may be useful for CRT prophylactics. Lastly, obesity for Chinese is defined as body mass index (BMI) weight ≥ 28 [5]. In general, the cephalic vein cannulation should not be considered as a routine procedure in ICU due to high anatomy variation, difficult process and time consuming. Where the ultrasound-guided center venous cannulation technique was mastered excellently, where the cephalic vein access can be considered on condition that sufficient evaluation is prepared.

## Data Availability

Not applicable.
